# Significance of Hyperreflective Foci as an Optical Coherence Tomography Biomarker in Retinal Diseases: Characterization and Clinical Implications

**DOI:** 10.1155/2021/6096017

**Published:** 2021-12-17

**Authors:** Serena Fragiotta, Solmaz Abdolrahimzadeh, Rosa Dolz-Marco, Yoichi Sakurada, Orly Gal-Or, Gianluca Scuderi

**Affiliations:** ^1^Ophthalmology Unit, Department NESMOS, S. Andrea Hospital, University of Rome “La Sapienza”, Rome, Italy; ^2^Unit of Macula, Oftalvist Clinic, Valencia, Spain; ^3^University of Yamanashi, Yamanashi, Japan; ^4^Department of Ophthalmology, Rabin Medical Center, Petah Tikva, Israel

## Abstract

Hyperreflective foci (HRF) is a term coined to depict hyperreflective dots or roundish lesions within retinal layers visualized through optical coherence tomography (OCT). Histopathological correlates of HRF are not univocal, spacing from migrating retinal pigment epithelium cells, lipid-laden macrophages, microglial cells, and extravasated proteinaceous or lipid material. Despite this, HRF can be considered OCT biomarkers for disease progression, treatment response, and prognosis in several retinal diseases, including diabetic macular edema, age-related macular degeneration (AMD), retinal vascular occlusions, and inherited retinal dystrophies. The structural features and topographic location of HRF guide the interpretation of their significance in different pathological conditions. The presence of HRF less than 30 *μ*m with reflectivity comparable to the retinal nerve fiber layer in the absence of posterior shadowing in diabetic macular edema indicates an inflammatory phenotype with a better response to steroidal treatment. In AMD, HRF overlying drusen are associated with the development of macular neovascularization, while parafoveal drusen and HRF predispose to macular atrophy. Thus, HRF can be considered a key biomarker in several common retinal diseases. Their recognition and critical interpretation via multimodal imaging are vital to support clinical strategies and management.

## 1. Introduction

The advent of optical coherence tomography (OCT) has dramatically changed the comprehension of pathophysiological mechanisms underlying retinal disease by detecting novel structural alterations *in vivo* [1]. The term “hyperreflective foci (HRF)” was coined to describe any hyperreflective lesion, focal or dotted in appearance, visualized on OCT at any retinal layer [2]. However, the clinicopathological correlate of HRF remains uncertain, ranging from lipid extravasation in diabetic macular edema (DME) [2], migrating retinal pigment epithelium (RPE) cells, macrophages/microglia in AMD [3–5], and degenerated photoreceptor cells [6].

The presence of HRF has revealed prognostic and clinical implications in several retinal diseases [7–12] and has influenced the evaluation of treatment response in DME [13,14]. In particular, HRF have been hypothesized to represent microglial cells when responding to specific morphometric criteria visible on OCT B-scans. Their characterization has improved the recognition of a preponderant inflammatory component that drives the management and treatment response of DME [15–18]. Beyond the established role of HRF as biomarkers in DME, their recognition and evaluation in various other retinal disorders might lead to a change in management, treatment, and prognosis.

The present study aims to provide an overview of the existing literature on HRF as OCT biomarkers associated with disease progression, treatment response, and prognosis of several retinal disorders, including DME, AMD retinal vein occlusion, retinal dystrophies, and uveitis.

## 2. Methods

A literature search of the Medline database was performed using the term “hyperreflective foci” for articles published in English, last accessed on 9^th^ December 2020. The articles selected described the clinical and prognostic implications of intraretinal and choroidal HRF detected in retinal diseases. Of 212 publications, 119 manuscripts published between 2005 and 2020 are reported in this narrative literature review. Reference lists of the selected manuscripts were also analyzed to retrieve other relevant studies.

## 3. Diabetic Retinopathy

### 3.1. Origin and Morphometry of Hyperreflective Foci

The presence of HRF in treatment-naïve DME was first described by Bolz et al. [2] as hyperreflective dots distributed throughout all the retinal layers, often within the septae between cystoid spaces, or confluent lesions located in the outer retinal layers, or focal deposits within the vascular wall of microaneurysms. These hyperreflective lesions were believed to represent extravasated protein and/or lipid deposits, precursors of hard exudates, that tended to resorb along with intraretinal fluid after laser treatment [2,19,20].

Another theory hypothesized that HRF were lipid-laden macrophages migrating into cystoid spaces as a consequence of blood-retinal barrier (BRB) breakdown [21,22]. However, with the introduction of OCT angiography (OCTA), it was noticed that some HRF presented decorrelation signals, possibly an expression of morphological changes in microglia/macrophages or intracellular organelles containing highly reflective material [23]. Glial cell proliferation represents one of the main alterations in diabetic retinopathy, and the role of microglia is essential to maintain retinal homeostasis and the inflammatory response [24].

Suspended scattering particles in motion (SSPiM) is a novel OCTA feature characterized by a flow artifact produced by moving material within cystic spaces possibly due to large molecules such as serum proteins and albumin permeated through the retinal interstitium [25]. SSPiM is closely related to the number of HRF and is considered the product of severe inner BRB breakdown. Accordingly, hyperreflective cystoid spaces, detected either on OCT B-scans or OCTA, often co-localize with HRF [25,26].

Combined multimodal analysis showed that HRF mainly occupy the outer nuclear layer (ONL) and outer plexiform layer (OPL) with distribution of the smallest foci in the inner nuclear layer (INL) and inner plexiform layer (IPL) and posterior shadowing caused by larger foci [27]. The identification of HRF on OCT B-scans demonstrated high interobserver reproducibility, comparable to other retinal OCT features detected in DME such as intraretinal fluid, diffuse retinal edema, subretinal fluid, and vitreomacular traction [28].

HRF cannot be identified with clinical examination because of their small size and axial thickness, and appropriate imaging resolution is necessary for their recognition. It was hypothesized that the foci gradually tend to grow and coalescence into visible lesions as hard exudates [27]. On near-infrared autofluorescence (NIR-AF), a patchy hyper-hypoautofluorescent signal described as a mosaic pattern was associated with the presence of HRF in the outer retinal layers and external limiting membrane (ELM) disruption and was considered a biomarker of photoreceptor damage [29]. Likewise, a granular appearance on both short-wavelength fundus autofluorescence (FAF) and NIR-AF was associated with the presence of HRF and visual impairment [29].

Interestingly, Lee et al. [30] demonstrated that the CD14 proinflammatory cytokine expressed by microglia, monocytes, and macrophages correlated with HRF, located in the inner retina, and diffuse edema. A nonobese diabetic mice model showed that proinflammatory cytokines induced both vitreal and retinal HRF and upregulated microglia cells [31].

**Figure 1:**
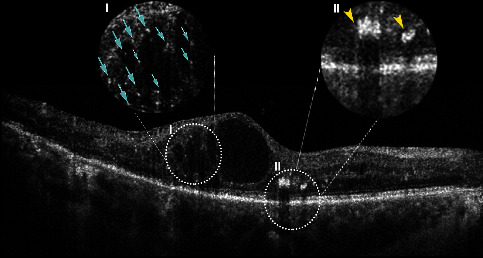
Spectral-domain optical coherence tomography B-scan showing the morphological differences between the inflammatory hyperreflective foci (Inset I is characterized by small size (≤30 *μ*m), the absence of posterior shadowing, inner and outer retinal location, and the reflectivity similar to the retinal nerve fiber layer (*light blue arrows*), and other subtypes of hyperreflective material (Inset II) such as retinal exudates are characterized by preferential location in the outer retinal layers, size > 30 *μ*m, the presence of posterior shadowing, and reflectivity similar to the retinal pigment epithelium (*yellow arrowheads*)).

The distinction between inflammatory HRF and other subtypes of hyperreflective material (i.e., retinal exudates, hemorrhages, and microaneurysms) on OCT B-scans include location within the inner retina, size ≤30 *μ*m, absence of posterior shadowing, and reflectivity similar to the retinal nerve fiber layer (Figure 1) [15,32,33]. Indeed, in a recent international consensus, these morphological characteristics were incorporated as the diagnostic criteria for HRF [17].

### 3.2. Clinical and Prognostic Implications of Hyperreflective Foci in Diabetic Macular Edema

The amount of HRF reflects disease severity, exhibiting direct associations with HbA1c values and high levels of total cholesterol, triglycerides, and low-density lipoprotein [34–37]. The association with glycometabolic state has been observed even in early stages of diabetic retinopathy without DME, supporting the hypothesis of lipid extravasation conceivable in subjects with poor glycemic control [38,39].

In eyes with DME, HRF located in the outer retinal layers have been strongly associated with worse visual prognosis, disruption of the ELM, photoreceptor loss, and worse prognosis after vitrectomy [6,29,40,41]. An alternative method of studying the integrity of the photoreceptor-RPE complex in the so-called “parallelism” supported that HRF in the outer retinal layers affects photoreceptor layer continuity. “Parallelism” is a term coined to evaluate retinal layer integrity through OCT B-scans postprocessing using dedicated software for imaging analysis. In brief, this parameter measures how straight the layers are and how parallel the layers are to each other [42]. The parallelism reflected the image complexity and the retinal structural changes, and it is lower in DME eyes than normal eyes. Parallelism also indicates the integrity of photoreceptors, exhibiting a direct association with visual acuity. One of the main factors affecting the parallelism with a relationship with photoreceptor integrity and visual function is the presence of HRF in the outer retinal layers [43,44].

The number of HRF in the outer retinal layers, as a predictor of final visual acuity, was associated with different patterns of DME including diffuse macular edema, cystoid macular edema, and serous retinal detachment [45]. The detection of similar HRF within the choroidal vasculature also denoted worse disease severity and prognosis [46,47]. In this regard, treatment-naïve DME with inflammatory biomarkers (i.e., HRF and serous retinal detachment) showed a superior anatomical response and fewer injections with a dexamethasone (DEX) intravitreal implant, even if better visual acuity was achieved with intravitreal aflibercept. Lens opacity development explained the lower-than-expected functional outcome in the DEX group [48]. A theoretical advantage in favor of a DEX implant as the first-line agent over anti-VEGF therapy has been hypothesized for DME with inflammatory biomarkers [17].

Changes in intraretinal HRF distribution during DME resorption after anti-vascular growth factor (VEGF) treatment included descending migration toward outer retinal layers, supporting the role of the osmotic gradient in fluid and macromolecule clearance [49]. DME with HRF has been associated with a poorer visual outcome following treatment with intravitreal steroid and anti-VEGF agents [14]. Clusters of HRF occupying the central macular area was associated with worse visual acuity than eyes without HRF clusters before any treatment, and the functional difference was maintained following intravitreal ranibizumab and focal laser therapy for up to 5 years [50].

While the role of HRF in predicting visual outcome of DME treated with anti-VEGF agents did not reach univocal conclusions [34,45,51,52], final visual gain resulted evident in DME eyes managed with DEX implant [13,53]. Treatment with DEX implant significantly modulated the number of foci with a reduction maintained up to 12 months of follow-up [18]. However, the reduction of the number of HRF located in the outer retina, modulated by anti-VEGF treatment, improved visual gain [54,55]. The prognostic role of HRF has been further corroborated by the higher levels of both IL-1*β* and HRF (>10) in refractory DME [56]; likewise, a high HRF number at baseline is predictive of early recurrence of DME and a shorter duration of DEX implant efficacy [57,58]. Patients with DME managed with observation exhibited a high risk of visual loss in the presence of DRIL, HRF, and ellipsoid zone disruption at baseline [11].

Evidence of HRF in the foveal region influenced postoperative visual recovery in eyes with vitreous hemorrhage due to proliferative diabetic retinopathy [59]. Nevertheless, their presence seemed to be independent of macular and peripheral retinal ischemia [60].

Recently, multiple (more than 30 in number) and diffuse HRF were considered integrant criteria of severity in the OCT grading proposed for diabetic maculopathy by an international panel of retinal experts [16].

## 4. Age-Related Macular Degeneration

### 4.1. Pathogenesis and Imaging Characterization

Khanifer et al. first reported HRF in AMD in 2008 [61] and analyzed drusen ultrastructure with spectral-domain (SD) OCT. Interestingly, the presence of HRF was noted overlying areas of RPE elevation and often in association with calcified drusen [61–63].

It was generally believed that HRF represent anteriorly migrating RPE cells and possible disaggregated photoreceptors, as supported by the corresponding pigmentary changes visible on color photographic images [61,64,65]. However, hyperpigmentation is not detectable in all cases [61], opening different hypotheses for a non-RPE origin. The foci may represent microglia migrating from the inner to the outer retinal layers engorged by lipid droplets or cholesterol [3,4]. This alternative HRF population has variable morphological characteristics such as size, migration, and clumping. Furthermore, microglial activation was particularly related to neovascular disease as validated through histopathology [4,66].

**Figure 2:**
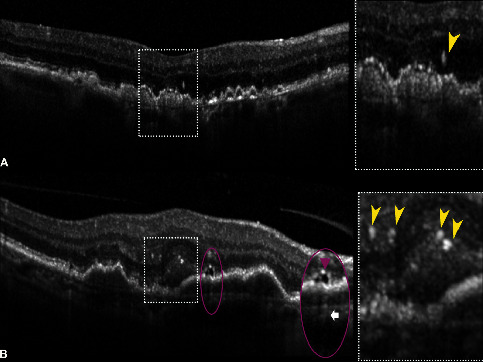
Spectral-domain optical coherence tomography (SD-OCT) B-scans illustrating hyperreflective foci (HRF) in age-related macular degeneration (AMD). (a) SD-OCT B-scan of intermediate AMD demonstrating a large HRF overlying confluent drusen located just above the external limiting membrane within the outer nuclear layer (inset, *yellow arrowhead*). HRF may represent migrating retinal pigment epithelium cells or a nascent type 3 lesion. Nascent lesions can be differentiated from type 3 macular neovascularization for the absence of exudative changes, as intraretinal fluid and cystic changes on OCT B-scans. (b) SD-OCT B-scan showing a case of macular neovascularization with multiple HRF located in the subretinal space and outer plexiform layer (inset, *yellow arrowheads*), probably of microglial origin, and more interestingly associated with a subretinal lipid globule. This novel OCT feature appears as a roundish hyporeflective structure (inset, *purple ellipse*) with a characteristic hypertransmission tail (*white arrow*), which is originated from a lensing effect produced by a lipidic content [74].

HRF located above the external limiting membrane and ONL/OPL junction, often co-localized with a drusenoid pigment epithelial detachment (PED), can also represent the antecedents of type 3 macular neovascularization (MNV), or the so-called *nascent* type 3 [67–73]. Nascent type 3 lesions were described as associated with HRF located within the ONL, OPL, or INL on OCT B-scans with a detectable flow signal on OCTA but without evident exudation (e.g., intraretinal fluid and microcystic changes) [67].  Figure 2 illustrates the OCT appearance of HRF in the context of intermediate AMD and MNV [67,74].

Intraretinal HRF from a possible RPE source have been characterized on clinicopathological correlations as isolated or grouped pigmented, nucleated RPE cells that shadow posteriorly on OCT B-scans often associated with hypertransmission areas reflecting the atrophic and dissociated RPE cells [3,75,76]. Different RPE histological phenotypes corresponding to hyperreflective structures on OCT B-scans were described [3,75]. Among these phenotypes, the RPE plume denoted a peculiar OCT feature with a comma-shaped configuration of HRF, believed to represent grouped migrating RPE cells within the Henle fiber layer [3,75].

### 4.2. Clinical Relevance and Prognostic Implications

HRF can be detected in intermediate to advanced AMD, demonstrating a predictive role for AMD progression and prognostic value when macular complications occur [77–80]. HRF were associated with disease severity, particularly in eyes with intermediate AMD, where they tended to increase in number and density and migrated from the ONL to the inner retinal layers over time [9,77,81]. In intermediate AMD, retinal sensitivity assessed through microperimetry was affected by the presence of HRF that typically co-localized with alterations of the outer retinal bands and the RPE [82–84]. HRF represented markers of cellular dysfunction responsible for visual decline before the development of macular complications [85]. Hyperreflective specks (HRS) shared similar features with HRF, appearing as hyperreflective dots preferentially located in the Henle fiber and ONL associated with visual dysfunction. HRS distinctive features included smaller diameter, lower reflectivity than the RPE band, and more uniform size than HRF. Both HRF and HRS were considered markers of cellular activity, with HRS representing lipofuscin granules, translocated inwardly within cone photoreceptors [85].

More interestingly, the increasing number of HRF was associated with RPE atrophy and considered a precursor of geographic atrophy [64,77,79]. Several factors have been implicated in macular atrophy progression, including drusen volume, HRF, HRF within a drusenoid lesion, and subretinal drusenoid deposits [86,87]. However, HRF represented the strongest predictor alone for progression to both central or any geographic atrophy [86,88,89]. In progression of geographic atrophy, the morphological features accompanying the presence of HRF were often characterized by reduced retinal thickness and volume and ONL thinning [90]. The distribution of HRF varied according to the subtype of macular complication. Eyes developing macular atrophy presented HRF co-localizing with drusen at 0.5 mm eccentricity, not at the foveal center [91]. Deep learning quantification of HRF in late AMD demonstrated their spatial localization at the atrophy border, demarcating areas subject to growth and expansion of existing atrophic lesions. Furthermore, HRF tended to accumulate in correspondence to areas developing de novo lesions [92].

Similarly, in eyes with drusenoid PED, the presence of HRF at baseline and their migration throughout retinal layers were directly associated with new-onset atrophy [78]. Changes in HRF preceded drusenoid PED collapse, where migrating RPE cells and subsequent RPE disintegration, responsible for hypertransmission, accompanied the PED breakpoint [93].

The prognostic role of HRF has been proven for predicting neovascular conversion. Both the presence and HRF count represented strong predictive biomarkers of neovascular progression [7]. Precursors of type 3 lesions were typically represented by HRF located in the outer retinal layers [67,72,94]. One of the possible mechanisms underlying neovascular complications was represented by increased choriocapillaris ischemic changes found to be more severe in eyes with HRF [95]. The predictive value of HRF was mostly associated with drusen growth accompanied by overlying HRF in MNV conversion, suggesting a distinctive hallmark of neovascular conversion [90,91,96].

In eyes with MNV, HRF were diffusely distributed in the neurosensory retina and their presence was associated with a poor visual outcome despite anti-VEGF treatment [97,98]. Anti-VEGF switching from ranibizumab to aflibercept demonstrated a morphological and functional improvement, including HRF reduction, associated with a decreasing central subfoveal thickness [8,99,100]. Of note, the presence of HRF was strongly correlated with intraretinal fluid [101]. HRF detection in neovascular AMD and polypoidal choroidal vasculopathy (PCV) was considered a reliable predictor of poor visual prognosis after anti-VEGF treatment [102].

## 5. Miscellaneous

The role of HRF has been investigated in other retinal vascular diseases, including branch retinal vein occlusion (BRVO) and central retinal vein occlusion (CRVO). In this regard, two distinct HRF populations have been identified, including fine scattered HRF probably related to extravasation of blood constituents and confluent HRF mainly located in the unaffected areas spared by the retinal occlusion. Confluent HRF were thought to be associated with the absorption of water and other molecules [103]. While fine scattered HRF cannot be visualized on fundus photographic images, confluent HRF were believed to represent retinal exudates [104]. In retinal vein occlusion, HRF were topographically scattered along the OPL and the external limiting membrane [103,105]. Similar to other retinal diseases, a poor visual outcome after anti-VEGF treatment accompanied the identification of HRF at baseline [104,106]. Furthermore, the use of intravitreal DEX implants might be privileged in eyes with numerous HRF and long-standing macular edema secondary to RVO in consideration with the inflammatory component [107].

**Figure 3:**
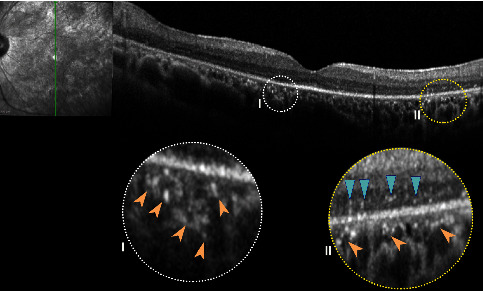
Spectral-domain optical coherence tomography B-scans showing an illustrative case of retinitis pigmentosa characterized by both choroidal hyperreflective foci (insets, *peach arrowheads*) and intraretinal hyperreflective foci (inset II, *light blue arrowheads*).

Among degenerative retinal diseases, retinitis pigmentosa (RP) revealed HRF with specific topographic distribution and association with disease progression. Eyes with HRF distributed in the INL denote an early stage of RP with spared RPE-Bruch's membrane complex. However, eyes with HRF in the ONL designate a more advanced disease characterized by photoreceptor loss and RPE cell migration and degeneration [108]. RPE cell degeneration in RP eyes occurs secondary to proliferation, spreading, and migration of the RPE cells toward the inner retina with bone spicule formation [109]. The distribution of HRF is mainly concentrated over regions of photoreceptor disruption and associated with intraocular inflammation, further corroborating the hypothesis of RPE or microglial migration in response to photoreceptor degeneration (Figure 3) [110]. Similarly, the recognition of choroidal HRF corresponded to ELM and ellipsoid zone disruption suggesting a migration of deteriorated photoreceptors and RPE cells from the outer retina toward the choroid due to a degradation process [111].

In Stargardt disease, the evidence of choroidal HRF primarily located in the choriocapillaris and Sattler's layer was considered a biomarker of disease severity in terms of atrophic changes and visual function [112,113]. Furthermore, the concentration of HRF was greater in atrophic areas measuring less than 5 mm^2^, hypothesizing that HRF tended to fade with atrophy enlargement [113].

In pathologic myopia, the HRF role has been investigated in myopic choroidal neovascularization and myopic macular hole [114,115]. HRF appeared to be associated with the presence of retinal edema, serous neuroretinal detachment, and hemorrhage in myopic choroidal neovascularization. All these signatures indicate an active retinal exudation, suggesting that HRF represent an additional indicator of choroidal neovascularization activity [114]. After myopic macular hole repair, the presence of HRF was associated with a worse visual acuity. The limited representation of HRF after macular hole repair with the inverted inner limiting membrane (ILM) flap technique was explained with superior sealing of the retina compartment, allowing the RPE to recover its pump function effectively [115].

HRF were recognized in uveitis and intraocular inflammatory disorders and were likely presumed to represent intraretinal exudates, lymphocytic cellular or clumping of photoreceptors or intraretinal RPE cells when related to photoreceptor loss [116–119]. In eyes with uveitic macular edema, HRF were associated with worse visual acuity [120]. After treatment, the foci decreased in number and mainly remained located to the inner retina layers [121].

## 6. Conclusions

Hyperreflective foci represent a univocal OCT feature revealing several possible histopathological correlates, including migrating RPE cells, microglia, precursors of exudates, or intraretinal neovascularization in the setting of AMD. HRF represent an important OCT biomarker with significant clinical and prognostic implications embracing several common macular diseases. The detection of HRF of size ≤30 *μ*m without posterior shadowing and reflectivity similar to the retinal nerve fiber layer configures the inflammatory phenotype in DME that usually responds better to early intravitreal steroid implant.

Relevance as a biomarker is also observed in AMD, where the number and distribution of HRF may be predictors for progression to advanced stages of disease. The co-localization of HRF overlying drusen associated with drusen growth in the foveal center is believed to be a predictor of neovascular progression. In contrast, a high concentration of HRF distributed at 0.5 mm of eccentricity edging the foveal pit, in the absence of drusen occupying the foveal center, tends to predispose to macular atrophy. Moreover, the presence of HRF influences anti-VEGF treatment response and visual prognosis of MNV. In conclusion, HRF can be considered a critical OCT feature with substantial predictive value for disease progression and treatment response in the principal macular disorders encountered in routine clinical practice. Their prompt recognition and critical interpretation may guide clinical and therapeutic strategies.

## Data Availability

Data are available upon request to the corresponding author Serena Fragiotta, MD, PhD, via e-mail (serena.fragiotta@uniroma1.it).
